# Selenium Species: Current Status and Potentials in Cancer Prevention and Therapy

**DOI:** 10.3390/ijms20010075

**Published:** 2018-12-25

**Authors:** Heng Wee Tan, Hai-Ying Mo, Andy T. Y. Lau, Yan-Ming Xu

**Affiliations:** Laboratory of Cancer Biology and Epigenetics, Department of Cell Biology and Genetics, Shantou University Medical College, Shantou 515041, China; hwtan@stu.edu.cn (H.W.T.); 16hymo@stu.edu.cn (H.-Y.M.)

**Keywords:** selenium species, Se-containing nanoparticles, anticancer, chemotherapeutics, epigenetics

## Abstract

Selenium (Se) acts as an essential trace element in the human body due to its unique biological functions, particularly in the oxidation-reduction system. Although several clinical trials indicated no significant benefit of Se in preventing cancer, researchers reported that some Se species exhibit superior anticancer properties. Therefore, a reassessment of the status of Se and Se compounds is necessary in order to provide clearer insights into the potentiality of Se in cancer prevention and therapy. In this review, we organize relevant forms of Se species based on the three main categories of Se—inorganic, organic, and Se-containing nanoparticles (SeNPs)—and overview their potential functions and applications in oncology. Here, we specifically focus on the SeNPs as they have tremendous potential in oncology and other fields. In general, to make better use of Se compounds in cancer prevention and therapy, extensive further study is still required to understand the underlying mechanisms of the Se compounds.

## 1. Introduction

Selenium (Se) is an essential micronutrient for the human body that is mainly obtained through diet and/or nutritional supplement [1]. Trace amounts of Se are required for maintaining optimal health as Se is a component of the selenoproteins (mostly in the form of amino acid selenocysteine) that participate in a wide range of cellular physiological processes. These processes include, but are not limited to, thyroid hormone regulation [2], redox homeostasis [3,4,5], inflammatory and immunological responses [6,7,8], carbohydrate metabolism [9], cardiovascular [10] and reproductive [11,12] health, and brain function maintenance [13,14,15]. Se deficiency is associated with numerous human diseases with various degrees of illnesses [16,17]. For instance, the Keshan disease (fatal cardiomyopathy due to viral infection) [18] and Kashin-Beck disease (chronic osteochondropathy) [19] are a few typical examples of Se deficiency-related diseases, which often occur endemically in the population living in regions with Se-poor soil. Excessive Se can be toxic and may lead to selenosis [17,20]. Currently, the recommended dietary allowance of Se for adults is set at 55 μg (0.7 μmol)/day [21]. Individuals with daily Se intake less than ~15 μg appear to be at risk of Se deficiency-related diseases, whereas those who consume over 400 μg/day are prone to Se toxicity, although some studies have shown that safe levels of Se intake may be much lower than anticipated [16,21].

The relationship between Se and cancer, particularly in gastrointestinal and prostate cancer, was discovered in the middle of the 19th century, which then raised interest in the contribution of Se supplements to cancer prevention and therapy [22,23,24]. During half a century of exploration, many novel forms of Se compounds have been discovered and tested, and some have shown promising anticancer activity [25,26]. A double-blind, placebo-controlled, and randomized clinical trial carried out in the 1990s, the Nutritional Prevention of Cancer (NPC) trial, has provided us the early evidence supporting Se as a potential chemopreventive agent [27,28,29]. However, to date, none of the Se compounds have been clinically recognized as anticancer drugs, partly because, over the years, researchers have obtained conflicting results within and between epidemiological, clinical, and laboratory studies [30,31]. Notably, in contrast with the NPC trial, subsequent clinical trials such as the Selenium and Vitamin E Cancer Prevention Trial (SELECT) failed to demonstrate the anticancer effects of Se [32,33,34]. Some of these conflicting results showed that Se compounds not only failed to exert their cancer prevention or anticancer ability as anticipated but, in some cases, may even promote cancer [35]. Recent epidemiologic evidence suggests that chronic exposure to inorganic Se may increase cancer risk [36]. As a result, the dual role of Se compounds in carcinogenesis, especially in relation to the aspects of oxidative stress and angiogenesis, has been proposed and recently summarized [31]. So far, there is no clear conclusion on the circumstances under which a particular Se compound prevents or enhances carcinogenesis, perhaps due to the wide variety of Se speciations and their diverse effects at different concentrations on different metabolic pathways of cells and tissues [37,38]. Se or Se-containing compounds can be grouped into three main categories: inorganic, organic (also known as the organoselenium compounds), and Se-containing nanoparticles (SeNPs). In order to better utilize the anticancer properties of Se species, it is necessary to thoroughly evaluate the current status of Se species. Here, we systematically organize the relevant forms of Se species, with slightly more emphasize on SeNPs, and review their recent developments and potential in cancer prevention and therapy.

## 2. Anti- or Pro-Cancer?

Several Se compounds derived from all the three groups of Se (inorganic compounds, organoselenium compounds, and SeNPs) have shown possible anticancer ability. It is generally accepted that Se compounds exert their anticancer ability mainly through their direct or indirect antioxidant properties that intracellularly maintain the redox status and protect healthy cells from reactive oxygen species (ROS)-induced oxidative damage [39]. ROS are free radicals with unpaired electrons generated during normal biophysiological function. The evidence is strong that excessive ROS promotes carcinogenesis via elevated oxidative stress and increased DNA mutation [40]. Cancer cells are often characterized by their ability to produce and cope with an increased amount of ROS [41]. In other words, increased dependence on an antioxidant defense system is one of the principal characteristics of cancer cells. Despite the links between ROS and cancer formation, however, optimal (usually low) levels of ROS are actually beneficial as they play important roles in regulating many biological functions. Some enzymes and cells (e.g., white blood cells) can deliberately produce a range of superoxide radicals to kill invading pathogens [39,42]. ROS can also destroy damaged cells by promoting cellular senescence and apoptosis and thus eliminate the formation of cancer [41,42]. Such a dual role of ROS may explain why conflicting results for Se species, as “antioxidants”, are often observed in cancer research. To further complicate the situation, other research found that some selenoproteins could actually behave as prooxidants instead of antioxidants, demonstrating both cancer-inhibiting and -promoting features in a cell type-, genotype-, and dosage-dependent manner [43,44,45,46,47]. For example, thioredoxin reductase 1, an essential redox regulating selenoprotein, can change from an anti- to a pro-oxidant and can both inhibit or promote carcinogenesis [44,46].

In addition to oxidative stress regulation, the duality of Se compounds on angiogenesis has been discovered [31,38]. Angiogenesis refers to the physiological process responsible for the formation and growth of micro-blood vessels from pre-existing vasculature, which is one of the most important mechanisms for cells to obtain oxygen and nutrients. The roles of angiogenesis in relation to cancer development and metastasis have been studied extensively, and therapy explicitly targeting angiogenesis has become a promising approach for cancer treatment [48]. In vitro and in vivo studies showed that some Se compounds, such as the monomethylated Se amino acid methylselenocysteine (MSC), could inhibit cancer growth through its antiangiogenic properties [49,50,51]. MSC might also normalize the blood vessels and thus enhance delivery of a range of chemotherapeutic drugs and simultaneously reduce their toxicity [52,53,54,55,56]. Conversely, opposite results in which pro-angiogenic responses of Se-selenoproteins/compounds in normal or cancer cells have also been reported [57,58]. Thus, the dual role and narrow window between the beneficial and toxic effects of Se compounds often limit their potential for clinical application.

The dual effect of Se mentioned in this section is often restricted to the inorganic and organic Se compounds, and so far, research on anticancer activity of SeNPs, the emerging special form of Se species, appears to be positive. The use of SeNPs has had a revolutionary impact on cancer therapy, and they have shown tremendous potential compared to “ordinary” inorganic and organic Se compounds [59,60,61,62]. However, knowledge regarding the cytotoxicity and other possible adverse effects of these SeNPs in humans is still lacking, and further extensive research is required [63,64]. So far, all Se compounds are considered non-carcinogenic with the exception of selenium sulfide, which is categorized as a probable human carcinogen. Overall, in order to determine the potential of Se compounds in cancer prevention and therapy, multiple factors (e.g., speciation, concentration, targeting cell type, and cell state/condition) must be considered. In the following sections, we organize relevant forms of Se species and discuss their potential roles in cancer treatment based on recently published data.

## 3. Se-Containing Compounds and Their Usage in Oncology

All three main categories of Se (inorganic, organic, and SeNPs) contain compounds with potential anticancer properties. For inorganic and organic Se compounds, research has found that they are both metabolized differently and have varied mechanisms of action in diverse bio-physiological processes, including their roles in cancer [65]. Both forms of Se compounds can be readily absorbed by the human body, but only organic Se compounds, usually in the forms of amino acids (e.g., selenomethionine (SeMet) and selenocysteine), can be better retained and used [65]. The cancer prevention ability of a range of inorganic and organic Se compounds has been supported by a large number of publications from a wide range of studies under different settings, including biochemical, epidemiological, clinical, and animal studies [38,44,46,66,67,68]. However, toxicity risks accompanied by the use of these Se compounds have also been recorded. Although organic forms of Se may have lesser toxic effects than inorganic Se compounds [69], in reality, the toxic effects of Se are determined by multiple factors, with the forms of Se and dosage exposure being two of the most important parameters [26]. Despite the greater toxic effects, inorganic Se compounds may have an advantage in certain aspects of cancer therapy as described below.

### 3.1. Inorganic Se Compounds

**Figure 1:**
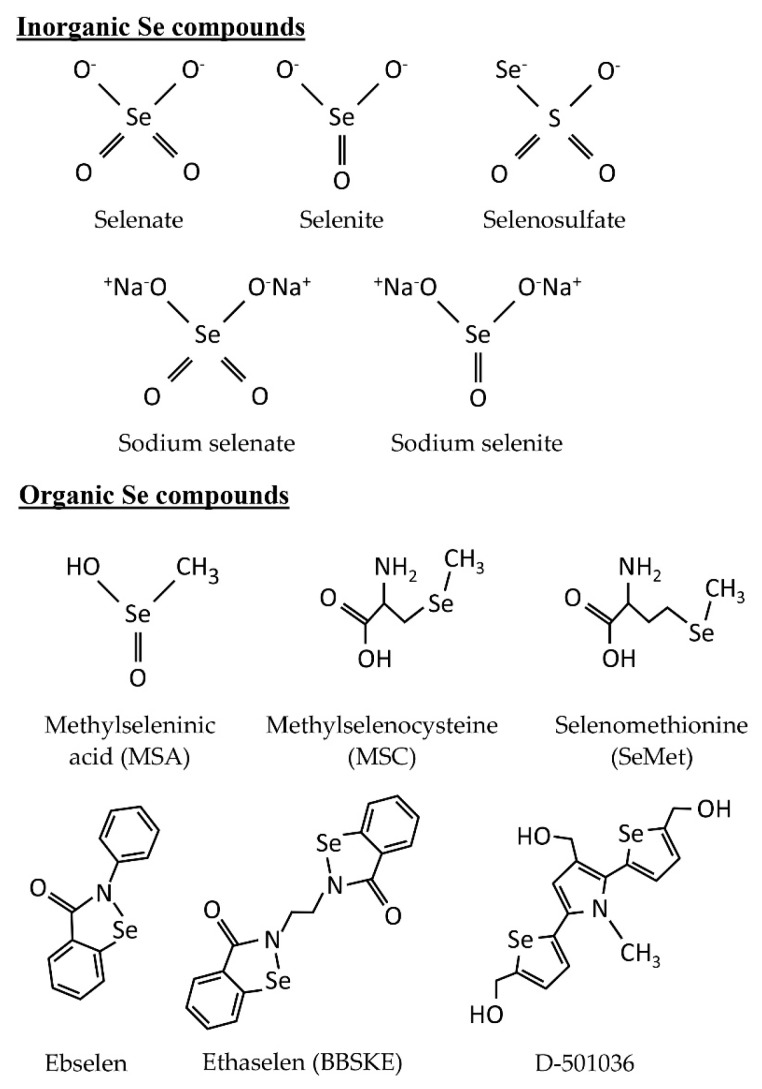
Chemical structures of selected representative inorganic and organic Se compounds discussed in this review.

Se exists in four natural valence states: elemental Se (0), selenide (−2; Se^2−^), selenite (+4; SeO_3_^2−^), and selenate (+6; SeO_4_^2−^). In Figure 1, we display the chemical structures of some of the representative Se compounds. A more detailed list of classification of Se compounds based on their structural features is summarized by Sanmartín et al. [70]. The functional and toxic effects of inorganic Se compounds differ according to their valence states. In Choi et al. [71], various concentrations of sodium selenate (Na_2_SeO_4_) (5, 10, 30, and 50 µM for 48 h) and sodium selenite (Na_2_SeO_3_) (0.1, 0.25, and 0.5 µM for 48 h) along with three other organic Se compounds (SeMet, MSC, and methylseleninic acid (MSA)) were tested for their ability to sensitize human oral squamous carcinoma (KB) cells resistant to chemotherapeutic drug vincristine (KBV20C). They found that although all five Se compounds appeared to be able to sensitize KBV20C to the same extent as the sensitive parent KB cells, only selenate produced a higher sensitizing effect on the KBV20C cells by arresting the cell cycle at G2-phase and activating apoptotic pathways. However, opposite results were obtained by Takahashi et al. [72], where they showed that human oral squamous carcinoma (HSC-3) cells were more sensitive to Na_2_SeO_3_ and selenium dioxide (SeO_2_), but not sodium selenate at concentrations ranging from 1 to 1000 µM (72 h). Pronounced anti-proliferative effect of selenite (5–100 µM for 2–5 days) against three oral cancer cell lines (HSC-3, HSC-4, and SAS) was reported [73]. This study also suggested that selenite had a better anticancer effect than the two other organoselenium compounds (SeMet and MSC) tested.

Selenite is the most studied form of inorganic Se compounds as it exhibits excellent chemopreventive and anticancer features [74]. Selenite could effectively inhibit cell proliferation of various types of cancer cells, including lung cancer, which is the most common and deadliest cancer worldwide [75]. Among different human cancer cell lines tested, lung cancer cells, in general, appeared to be especially sensitive to selenite [76,77,78,79]. Olm et al. [80] indicated that selenite cytotoxicity (5 µM for 5 h) was correlated with Se uptake of three lung cancer cell lines (H157, H611, and U2020) and that high concentrations (>1 mM) of selenate were non-toxic for these cell lines. Selenite was suggested to play a role in natural killer (NK) cell-based anticancer immunotherapy where it could increase the susceptibility of cancer cells to CD94/NK group 2A-positive NK cells, and has possible clinical applications in lung cancer patients [81]. The synergistic effect of selenite and thioredoxin reductase inhibitors (e.g., ethaselen and auranofin) was detected in human ovarian and lung cancer cell lines [82,83]. These results demonstrated the potential of Se compounds to enhance the activity and reduce the toxicity of anticancer drugs including those commonly used in chemotherapy (e.g., cisplatin, docetaxel, 5-FU, oxaliplatin, and irinotecan) [84,85,86]. Se compounds appear to be more effective in inhibiting the growth of anticancer drug-resistant cancer cells compared with drug-sensitive cancer cells via deactivating various resistance mechanisms used by the cancer cells. Chemotherapeutic drug-resistant lung cancer cells were revealed to be generally more sensitive to selenite (ranging from 0.1 to 100 µM for 48 h and up to 4 days) compared to drug-sensitive cancer cells [82,87,88]. In addition to the above-mentioned in vitro studies, a phase I clinical trial published demonstrated the beneficial effects of selenite in cancer patients, especially in lung cancer patients who were resistant to cytostatic drugs [76].

Notably, the above results, however, do not necessarily mean that selenite is superior to other inorganic Se compounds in terms of cancer prevention and therapy. For instance, selenate might be more effective than selenite under certain circumstances as described earlier, even in the same type of cancer albeit different cell lines [71,72]. Inorganic Se such as selenosulfate (SeSO_3_^−^) was reported to have generally greater cytotoxic effects on cancer cells but was less toxic in healthy cells than selenite, depending on the cell types and the presence or absence of supplement amino acids that may affect the uptake of selenite [89]. Both SeSO_3_^−^ and selenite might have the potential to work as a remedy against chemotherapy toxicity [76,84]. It was shown that hydrogen selenide (H_2_Se), a common intermediate of dietary Se metabolism produced by reduced selenite, could trigger the apoptosis of cancer cells (HepG2, HeLa, and MCF-7 cells) via accumulation in mitochondria induced by selenite, which would subsequently damage mitochondrial function and structure and lead to cell death [90].

### 3.2. Organic Se Compounds

Organic Se compounds attract considerable attention in the field of cancer research mainly due to their lower toxicity risk and ability to deliver significant anticancer activity, comparable or sometimes even better than inorganic Se compounds [69]. Organic Se compounds can be classified into different families based on their functional chemical structures: Selenides/diselenides, selenocyanates, selenoaminoacid derivatives (e.g., SeMet and MSC), methylseleninic acid (MSA; CH_3_SeO_2_H), Se-heterocyclic compounds, and other miscellaneous Se-containing compounds (Figure 1). These organoselenium compounds exhibit anticancer and chemopreventive activity through diverse mechanisms of action, including reduction of oxidative stress [91], induction of apoptotic events [92,93,94], and enhancement of chemotherapeutic drug activity [95,96,97]. To date, many scientific studies on organoselenium compounds is available, many of which investigated their roles in cancer prevention and therapy. Several excellent reviews have listed a range of organoselenium compounds based on different classifications and summarized their functions in oncology [31,38,70,98]. Additionally, the potential anticancer and chemopreventive activity of selenides/diselenides [99] and selenocyanates [100] have been extensively reviewed.

Organoselenium compounds have the potential to be used as anti-neoplastic agents against solid tumors. Since necrosis of cancer cells is linked to host inflammatory response and may lead to treatment complications, the anti-necrotic and pro-apoptotic feature of some organic Se compounds is therefore largely preferred in cancer therapy [101]. Selenoaminoacid derivatives, such as SeMet and MSC, at low concentrations (as low as 0.113 µM), were shown to be able to promote apoptosis in solid tumors of various types of human cancer, whereas the control non-tumorigenic mammary epithelial cells (MCF-10A) required substantially high concentrations (up to 87.9 µM for 72 h) of organic Se to display sensitivity to apoptosis [92]. Owing to the ability of organoselenium compounds to induce apoptosis, their synergistic effects on chemotherapeutic drugs against cancer were observed [53,95,96,97]. MSC may also provide additional protection from the toxicity of anticancer drugs [54,56].

MSA is an oxidized form of methylselenol (CH_3_Se^−^) converted from selenoaminoacids (e.g., SeMet and MSC) [43]. In vivo and in vitro studies have indicated that MSA is an excellent anticancer agent comparable to SeMet or selenite against a range of cancer models, including lung [102], breast [103,104], melanoma [105], and prostate cancer in particular [106,107,108]. In a recent study, MSA showed significant cytotoxic effects toward monocytic leukemia cells (THP1) compared with the healthy peripheral blood mononuclear (PBM) cells [109]. MSA also enhanced the anticancer activity of radiation and chemotherapeutic drugs (cytosine arabinoside and doxorubicin) in the malignant THP1 cells in a dose-dependent manner (2.5, 5, and 15 µM for 48 h) [109]. At lower concentrations, MSA protected normal PBM cells from radiation and chemotherapeutic drugs, whereas at higher concentrations, MSA was considered toxic and could increase the cytotoxicity of radiation but not chemotherapy [109]. In another study, MSA was able to inhibit the proliferation, migration, and adhesion of HeLa cells more effectively than SeMet and MSC, and it showed synergistic anticancer activity with S-adenosyl-methionine—a universal methyl group co-substrate involved in multiple intermediary metabolites [110]. MSA was shown to be able to reverse the tamoxifen resistance of breast cancer cells when used in combination with tamoxifen through the activation of caspase-9 and then caspase-8, resulting in the induction of the intrinsic, mitochondrial apoptotic pathway [111]. A novel programmed cell death mechanism (entosis) induced by MSA and methylselenoesters was identified in pancreatic cancer Panc-1 cells [112]. Entosis is characterized by the invasion of a living cell to another cell’s cytoplasm resulting in endophagocytosis and the formation of cell-in-cell structures.

Other groups of selenocompounds, such as the selenocynates and Se-containing heterocycles, also contained several Se compounds with promising chemopreventive or anticancer properties, ranging from the well-studied *p*-xylene selenocyanate and benzyl selenocyanate to the recently reported novel active compounds that combine the selenocyanate moiety with different heterocycles, quinones, or steroids [100]. Heterocyclic organoselenium compounds, such as ebselen and ethaselen (also known as BBSKE) are small molecules that have potential in cancer therapy [113,114]. A list of these heterocyclic compounds and their anticancer ability is summarized in Fernandes and Gandin [38] and Sanmartíin et al. [70]. The selenophene-based triheterocyclic derivative 2,5-bis(5-hydroxymethyl-2-selenienyl)-3-hydroxymethyl-*N*-methylpyrrole (D-501036) has received increasing attention due to its broad-spectrum anticancer activity against several human cancer cells in a dose- and time-dependent manner [115,116,117]. D-501036 selectively induces apoptosis and double-strand DNA breaks in cancer cells, and is especially effective against chemotherapeutic drug-resistant cancer cells with overexpression of P-glycoprotein/multidrug-resistant protein [116]. Further study has suggested that enhanced non-homologous end-joining DNA repair activity was involved in the development of D-501036-resistance in cancer cells [117]. Previously in a cisplatin-resistant prostate cancer model, the degree of drug-resistance was found to be associated with the oxidative system in the cells [118]. Thus, it was likely that cancer cells, and in particularly drug-resistant cancer cells, have higher Se uptake compared with benign cells due to the redox state of Se and the oxidative system of the cancer cells [118,119].

Previous clinical trials (e.g., NPC) have shown that dietary Se supplements (Se-enriched yeast) could reduce the risk of multiple cancers [27,28,29]. However, subsequent trials (e.g., SELECT and Southwest Oncology Group (SWOG) S9917), tested using SeMet, showed no such beneficial effects [32,33]. In a clinical trial, it was indicated that SeMet did not improve quality of life or survival outcomes of patients with head and neck squamous cell cancer undergoing concurrent chemoradiation [35]. These conflicting results may be partially explained by the selection of the Se compound administered: 200 µg/day of Se-enriched yeast in the NPC trial and 200 µg/day of SeMet in SELECT and SWOG S9917. Although SeMet is the major component of Se-enriched yeast, it is possible that other components in Se-enriched yeast (e.g., MSC) may be contributing to the overall chemopreventive effect of Se observed in the NPC trial. This speculation, however, cannot be verified since there were substantial batch-to-batch variations in specific organoselenium compounds in the samples of NPC yeast [32]. Additionally, no significant benefit in the prevention of second primary tumors was detected in a phase III clinical trial (ECOG 5597) that administrated 200 µg/day of Se-enriched yeast to patients with completely resected stage I non-small-cell lung cancer [34]. Despite these negative findings, most of these trials suggested that Se supplement was safe to consume. Thus, organic Se may be useful in cancer therapy but appears to confer no significant benefit in the prevention of cancer.

### 3.3. SeNPs

Since the late 1990s, application of nanotechnology in the bio-medical field has received extensive attention. Owing to their biocompatibility, biodegradability, and designability, nanomaterials are increasingly utilized in cancer therapy and diagnosis as pharmaceutical products, drug carriers, imaging agents, and diagnostic reagents to overcome some limitations of the traditional materials [120]. SeNPs, as emerging Se species, are considered to be promising medical materials, according to their reported chemotherapeutical properties [121,122], nutritional effects [123], and relatively low toxicity compared with some other Se compounds [3,124]. The chemotherapeutical potency of SeNPs and their proposed anticancer mechanisms have been reviewed by Menon et al. [125]. Here, we systematically tabulated a list of SeNPs based on data published in 2017 and the first half of 2018 and arranged them according to their reported functions: chemotherapy (Table 1), drug delivery (Table 2), diagnosis (Table 3), and SeNPs with multiple functions (Table 4). The potential of these SeNPs in cancer prevention and treatment, along with their synthesis methods, are discussed below.

Many methods have been reported for SeNPs preparation, which are generally classified into three broad categories based on different producing principles: chemosynthesis, biosynthesis, and physical synthesis [159]. Among them, chemosynthesis is considered the most common method to prepare SeNPs. In chemosynthesis, Se in the +4-valence state, such as selenite, selenious acid, or SeO_2_, is frequently employed as precursors, whereas reducing agents (e.g., ascorbic acid and glutathione [GSH]) and stabilizing agents (e.g., chitosan and pectin) are used for SeNPs formation and maintenance [131,141,159,160]. SeNPs synthesized in the Na_2_SeO_3_-GSH redox system tended to gather in cancer cells and presented stronger pro-oxidant activity comparing to selenite [134]. However, to optimize their function in cancer therapy and prevention, chemosynthetic SeNPs are usually decorated with specific molecules, which endow them with ideal features to meet the demands of practical applications. For example, decorating SeNPs with other bioactive molecules can enhance the therapeutic effects upon certain types of cancers when compared with non-decorated SeNPs [128,129,161]. Aside from direct therapeutic effects, chemically modified SeNPs can function as vehicles that endow the carried objects with favorable properties like tumor targeting [150,157], high efficacy [137,144], and low toxicity [139]. Notably, chemosynthetic SeNPs were also examined as diagnostic agents [145,147,149], imaging agents [146,153], and radiosensitizers [130,158]. Overall, chemosynthesis is the most common method used to obtain and modify SeNPs, because the process is easy to implement and control. Nevertheless, environmental pollution and accumulation of chemosynthetic materials in the body should also be considered.

In contrast to chemosynthetic SeNPs, biosynthetic SeNPs appear to be environmentally friendlier and biologically safer. Thus, there has been increased focus upon biosynthetic SeNPs, partly also due to their extraordinary biocompatibility, sustainability, and economy [162]. These organismal materials mediating SeNPs are extracellularly or intracellularly manufactured with selective plants [64], bacteria [4], fungi [163], and other organisms [163,164]. Taking biological extracts as ingredients, researchers have successfully synthesized some chemical pollution-free SeNPs that display diverse biological effects, such as UVB-induced DNA damage prevention [165] and cancer cells proliferating inhibition [126]. So far, bacteria are the most important source of biosynthesized SeNPs. Bacteria such as *Bacillus licheniformis* JS2 [127], *Ochrobactrum* sp. MPV1 [166], *Streptomyces minutiscleroticus* M10A62 [167], and *Acinetobacter* sp. SW30 [126] have been employed to fabricating SeNPs. These bacteria-based SeNPs are synthesized by culturing bacterial strain with sodium selenite (0.5–2 mM). Zonaro et al. [166] demonstrated that, under the stress of Na_2_SeO_3_ (0.5 or 2 mM), *Ochrobactrum* sp. MPV1 was capable of converting selenite to elemental Se and synthesizing SeNPs intracellularly; however, medical applications of these SeNPs remain underexploited. The synthesizing protocol of bacteria-based SeNPs is now gradually developing; however, SeNPs should always be purified to avoid the toxicity caused by bacteria. Similar to bacteria, fungi with properties such as large output, easy accessibility, and economic feasibility could be used as candidates for biosynthesizing SeNPs [168]. However, the investigation into the practicability of fungi synthesized SeNPs in medical fields is relatively deficient. In Vetchinkina et al. [163], fungus-based spherical SeNPs were obtained from the medicinal basidiomycete *Lentinula edodes* cultured with inorganic selenium (10^−2^ mol) or organoselenium (10^−7^ to 10^−3^ mol). The reduction in selenium (Se^IV^ to Se^0^) was observed, and the synthesis mechanism of *L. edodes* based SeNPs was revealed by transmission electron microscopy, electron energy loss spectroscopy, and X-ray fluorescence [163]. Overall, bacteria and fungi are expected to be competent media for SeNPs assembling, given their capability of producing a hypotoxic form of Se by stepwise transforming Se oxyanions to elemental Se [169,170]. Intracellular synthesis of SeNPs could be achieved under ideal experimental conditions that usually require Se-involved culture [171], so that Se oxyanions would be transported into cells for downstream reduction and reassembly. In a study carried out by Sonkusre et al. [127], SeNPs synthesized in *Bacillus licheniformis* strain JS2 were able to initiate necroptosis in PC-3 cell by the ROS-mediated activation regulated by a RIP1 kinase, thus taken as undeveloped anti-cancer substances with high producing efficiency. Plant-based SeNPs are another interesting aspect of biosynthetic SeNPs [168]. For instance, Sharma et al. [64] synthesized selenium nanoballs with uniform size (3–18 nm) and shape using dried *Vitis vinifera* (raisin) extracts and selenous acid, with a simple refluxing method. Although the extract mechanism of plant-based SeNPs and the pharmaceutical applications remain largely underexplored, SeNPs synthesized with/in plants are likely to have vast potential for future development due to the variety of plants and their cleanliness.

As for the physically synthesized functional SeNPs, approaches such as pulsed laser ablation [172] and γ-radiation [173] have been used for their generation. For instance, Guisbiers et al. [174] obtained pure SeNPs by pulsed laser ablation in liquids and showed that these SeNPs were able to disturb the biofilm formation of a human pathogen *Candida albicans*, highlighting the potential for medical application of physical synthesis SeNPs. However, the application of physical-synthetic SeNPs in cancer treatment and prevention is still immature, which may be due to the equipment requirements.

From the above and Table 1, Table 2, Table 3 and Table 4, SeNPs have great potential not only in cancer treatment but also as diagnostic/imaging agents and more. Among all, the chemotherapeutic effects of SeNPs could be easily considered the most promising application of SeNPs. So far, results from laboratories regarding the anticancer property of SeNPs have mainly been positive. SeNPs showed anticancer effects in a range of cancers, including hepatocarcinoma [128,140], breast cancer [122,129,141], colon adenocarcinoma [124,132], lymphoma [133], esophageal cancer [143], prostate cancer [136,147], ovarian cancer [137], and glioma [148]. Further studies and clinical trials are needed to elucidate the possible applications of SeNPs in oncology. Also, concerns such as toxicity of nanoparticles accumulation in the human body and the environment should be cautiously addressed.

## 4. Se and Epigenetics: Possible Roles in Cancer Prevention and Therapy

Epigenetic refers to the study of heritable changes in gene expression that do not involve changes to the underlying DNA sequence [1,75]. These changes are controlled by epigenetic factors such as epimodifications of DNA, post-translational modification (PTM) of histone, and expression of non-coding RNA (ncRNA) [1]. Epigenetics plays a vital role in cancer development, and currently, therapy targeting epigenetic changes is considered one of the most promising approaches in cancer treatment [175,176]. The epigenetic effects of Se and their implications for human health, including cancer, have been reviewed [1,177,178]. Studies have revealed that Se and Se compounds could affect the epigenome of a cell through all three major epigenetic controls: DNA methylation, histone modifications, and ncRNA expression [1]. In regards to cancer therapy, inorganic and organic Se compounds, such as MSA, SeMet, MSC, Se-allylselenocysteine, and selenite, could effectively inhibit the activities of histone deacetylases and DNA methyltransferases, which expressions are usually up-regulated in various types of cancer cells [179,180,181,182]. Notably, the epigenetic inhibition mechanisms of Se compounds appear to be quite distinctive depending on their chemical forms [180,183]. A genome-wide epigenetic analysis indicated that both inorganic (selenite) and organic (MSA) Se could epigenetically affect distinct gene sets in human chronic myeloid leukemia K562 cells: selenite affected genes involved in response to oxygen and hypoxia, whereas MSA affected genes associated with cell adhesion and glucocorticoid receptors [184]. So far, the epigenetic effects of SeNPs and mechanism of their action on gene expression remain largely unknown.

## 5. Other Potential Applications of Se Compounds

Se is an essential trace element required for the maintenance of human health. In addition to its potential roles and use in cancer prevention and therapy, Se compounds have many other useful non-cancer-related features. Among them, one of the most interesting features of Se is the antimicrobial and antiviral activity observed in specific Se compounds. Previous studies demonstrated the relationship between selenocompounds and the immune system and showed that Se supplementation could enhance the activity and cytotoxic response of NK cells [6,7,8,38,81]. Specifically, sufficient intake of dietary Se is crucial for handling viral infections and for preventing Se deficiency-related diseases caused by bacteria and viruses (e.g., Keshan disease) [6,18]. Researchers have also discovered the antibacterial, antifungal, and antiviral properties of SeNPs, and have demonstrated how SeNPs can be potentially used in various settings [168,185,186]. For example, Nguyen et al. [63] examined the antimicrobial activity of SeNPs against foodborne pathogens and indicated the possible use of SeNPs for food safety applications. Overall, it is anticipated that new and novel applications of Se compounds in various fields of life sciences will be extensively investigated and explored due to rapid advancement in nanotechnology and further understanding of the mechanisms underlying Se compounds at the nano-scale level.

## 6. Conclusions

Although several clinical trials indicated no significant benefit of Se in preventing cancer, overwhelming evidence has demonstrated that Se and many Se compounds, under certain circumstances, are potent anticancer agents. In vivo and in vitro studies have hinted that the Se compounds exert their anticancer ability through multiple mechanisms. However, further research and clinical trials are still required before these Se compounds can be clinically recognized as anticancer drugs. In addition to cancer therapy, Se compounds have been proven to be quite useful in other cancer-related fields such as chemoprevention, diagnosis, and imaging, as well as in non-cancer-related fields as described in this review and summarized in Table 1, Table 2, Table 3 and Table 4. Among the Se compounds, SeNPs, as the emerging form of Se species, have attracted considerable attention. Judging from the current positive results of SeNPs against a range of cancers, SeNPs will play more critical roles in cancer prevention and therapy in the near future, especially in the era of precision medicine, where patients are provided with personalized and tailored treatment based on individual conditions and needs [75]. To conclude, in order to fully determine the potential of Se compounds, in particular the SeNPs, in cancer prevention and therapy, extensive further studies are required to better understand the underlying mechanisms behind the biophysiological effects of Se.

## Figures and Tables

**Table 1:** Summary of recent work on Se-containing nanoparticles (SeNPs) with potential in cancer chemotherapy.

SeNP	Material	Shape and Size (nm)	Effects	Dosage	Pathway	Model	Reference
Acinetobacter sp. SW30 SeNPs	Acinetobacter sp. SW30	Amorphous nanospheres, 78 nm; Polygonal-shaped, 79 nm	Selectively against breast cancer cells and non-toxic to normal cells	-	-	Breast cancer cells (4T1, MCF-7) and noncancer cells (NIH/3T3, HEK293)	[126]
Bacillus licheniformis JS2 derived biogenic SeNPs	Bacillus licheniformis JS2, aerobic condition in 1.8 mM Na_2_SeO_3_ stress	Spherical, 110 nm	Stimulated ROS production and caused damage to the mitochondria without affecting the cell membrane integrity. Induced overexpression of necroptotic genes and promoted RIP3-independent necroptosis	Concentration of 2 μg Se/mL	-	Human prostate adenocarcinoma cell line (PC-3)	[127]
Blg stabilized SeNPs	Ascorbic acid, Blg, Na_2_SeO_3_	Spherical, mean particle size of 36.8 ± 4.1 nm	Lower cytotoxicity than Na_2_SeO_3_. Similar cell growth inhibition on both colon cancer cell and corresponding normal cell	-	-	Human colon adenocarcinoma cells (HCT116) and colon normal cell (CCD112)	[124]
Ferulic acid-modified SeNPs	Na_2_SeO_3_, ascorbic acid, ferulic acid solution	Amorphous, average diameter of 109 nm	Induced intracellular ROS generation and MMP disruption	>100 μg/mL	Caspase-3/9, mitochondrial pathway	HepG2	[128]
Folic acid surface-coated Se nanoparticles (FA@-SeNPs)	Folic acid, SeO_2_ solution, ascorbic acid solution	Rod-shaped (400 × 100 nm)	Showed antiproliferative effect against 4T1 cells. Significantly increased the lifespan and reduced the tumor size of cancerous animals. Had better absorption toward cancer cells. Exhibited a better in vivo anticancer effect compared to SeNPs	200 μg/mL in vitro and 300 mg/week in vivo	-	4T1 breast cancer cell line and inbred Balb/c mice	[129]
Folic acid-conjugated SeNPs (FA@SeNPs)	Folic acid, CTS, Na_2_SeO_3_, ascorbic acid	Spherical, ~192 nm	Able to synergistically enhance the anticancer efficacy and colony formation inhibition ability of radioactive ^125^I seeds. Increased ROS overproduction. Induced DNA damage and activated the mitogen-activated protein kinase and TP53 signaling pathways	5 mg/kg of FA@SeNPs with an intratumor injection strategy every other day and/ or implanted with radioactive ^125^I seeds	DNA damage-mediated p53 and MAPK signaling pathways	Michigan Cancer Foundation-7 cell (MCF-7) and female nude mice	[130]
PEC-decorated Se nanoparticles	Selenite and ascorbic acid, PEC	Spherical, average size of ~41 nm	PEC as a surface decorator could be effectively used to improve the stability and antioxidant capacity of SeNPs	-	-	Cancer cells (SPCA-1 and HeLa) and normal cells (RWPE-1)	[131]
Pleurotus tuber-regium (PTR)-conjugated SeNPs (PTR-SeNPs)	Sclerotia of tiger milk mushrooms, Na_2_SeO_3_, ascorbic acid	Se concentration: 1.35 ± 0.12 μM; particle size: 80.0 ± 12.3 nm	Triggered intracellular G2/M phase arrest and apoptosis. Activated autophagy to promote the death of cancer cells	-	Beclin 1-related signaling pathways	Human colon cancer cells (HCT 116)	[132]
SeNPs	Se powder, Na_2_SO_4_, acetic acid	Spherical, 12–30 nm	Induced TAMs isolated from DL-bearing mice. Induced ROS generation, macrophage polykaryon formation, and adhesion molecules (CD54 or ICAM-1), and fusion receptors (CD47 and CD172α) expression on TAMs. Decreased tumor cell proliferation	20–50 ng for 10^6^ cells	-	Daltons lymphoma cells and DL-bearing BALB/c (H2d) strain of mice	[133]
SeNPs	SeO_2_	-	Combination of AET and SeNP supplementation effects anti-tumor immune responses in splenocytes	6 weeks of AET and SeNP administration (100 mg three times/week). Oral administration in doses of 100 and 200 mL per mouse	-	Mice bearing the 4T1 mammary carcinoma	[122]
SeNPs	Na_2_SeO_3_, GSH, BSA	20–70 nm, average size of 40 nm	Able to rapidly, massively, and selectively accumulate in cancer cells. Showed stronger pro-oxidant property than selenite	-	-	Male Kunming mice and Murine H22 hepatocarcinoma cells	[134]

Abbreviations, AET: aerobic exercise training; Blg: beta-lactoglobulin; BSA: bovine serum albumin; CTS: chitosan; DL: dalton’s lymphoma; MMP: mitochondrial membrane potential; PEC: pectin; ROS: reactive oxygen species; SeNPs: Se-containing nanoparticles; TAMs: tumor-associated macrophages; TrxR: thioredoxin reductase.

**Table 2:** Summary of recent work on SeNPs with potential for anti-cancer drug delivery.

SeNP	Material	Shape and Size (nm)	Effects	Dosage	Pathway	Model	Reference
CPT and DOX-loaded PEG-b-PBSe core crosslinked micelles (CPT/DOX-CCM)	Diselenide diols precursors, PEG45-based RAFT agent, CPT, DOX	Spherical, ~129 nm	Features include high drug loading, visible light-induced in situ crosslinking, improved physiological stability, optimized pharmacokinetics, and tumor-specific combined drug release	1 mg CPT or DOX equivalent per kg every four days in 24 days	-	Human breast cancer cell line (MCF-7) and mouse mammary tumor cell line (EMT-6)	[135]
CTS-modified Se nanoparticle	Na_2_SeO_3_, ascorbic acid solution (vitamin C), CTS	400–4000 cm^-1^	Slow-release carrier conjugated to the TNF-α-derived peptide P16, G0/G1 cell-cycle arrest, and apoptosis	-	p38MAPK/JNK pathway	Prostate cancer cells (DU145) and normal human prostate epithelial cells (RWPE-1)	[136]
DOX-SeNPs@TMC-FA (pH-sensitive)	Selenite, ascorbic acid, folic acid-N trimethyl CTS (TMC-FA)	An average diameter of 50 nm	Enhanced the activity of DOX by approximately 10-fold for a reduced IC_50_ value compared to free DOX	-	Apoptosis pathway involved caspase-3 and PARP proteins	Ovarian cancer DOX sensitive (OVCAR8) and resistant (NCI/ADR-RES) cells	[137]
EPI-loaded-NAS-24-functionalized PEIPEG-5TR1 aptamer coated SeNPs (ENPPASe complex)	Na_2_SeO_3_, EPI-loaded-NAS-24-functionalized PPA complex	68.2 ± 6 nm	Able to provide high loading of EPI and NAS-24. Reduced the toxicity in non-target cells. Reduced the cell viability in the target cancer cells. Reduced the tumor growth in cancer-bearing mice compared to EPI treatment alone	-	-	Human breast carcinoma cell (MCF7), murine colon carcinoma cell (C26) and human hepatocellular carcinoma cell (HepG2)	[138]
FA-CP/SeNPs	Na_2_SeO_3_, folic acid decorated cationic pullulan (FA-CP)	Flower-like structure, approximately 50 nm	Higher loading capacity of DOX. Less toxicity against normal cells	-	-	KB cancer cells line and normal cell line (L292)	[139]
Hyaluronic acid Se-PEI nanoparticle	Na_2_SeO_3_, ascorbic acid, hyaluronic acid, PEI	70–180 nm	Showed higher transfection efficiency, greater gene silencing ability, and stronger cytotoxicity	-	-	HepG2 cell, Lo2 cell and xenograft mouse model	[140]
Nano-Se + Nano-fluorouracil	Na_2_SeO_3_, GSH, BSA	Spherical, ranged from 66.43 nm to 98.9 nm	Induced chemo-sensitivity of 5-fluorouracil-encapsulated poly (D, L-lactide-co-glycolide) nanoparticles (nano-FU) in cancer cells	0, 2, 4, 6, 8, 10, 30, and 50 μM	Glucose uptake slight blockage, interaction with Zn	Human breast cancer (MCF7) and human colorectal cancer Cell (Caco-2)	[141]
Oil-soluble CdSe QD	CdO, mineral oil, oleic acid, Se	DG-PEG-OC-9R, near spherical, 112.0 ± 1.63 nm; FA-PEG-OC-9R, near spherical, 115.2 ± 1.94 nm	Could be used to evaluate the hypoxic tumor cell-targeting properties of the wrapped CTS-based micelles	-	-	Normoxic/hypoxic HepG2 and HeLa cells	[142]
Oridonin-loaded and GE11 peptide conjugated SeNPs (GE11-Ori-SeNPs)	Na_2_SeO_3_, oridonin, ascorbic acid, GE11 polypeptide	Near-spherical, average diameter of 70 nm	The GE11 surface modification provides targeting towards cancer cells: oridonin releasing induced cancer cell apoptosis. Inhibited tumor growth via inhibition of tumor angiogenesis by reducing the angiogenesis-marker CD31 and activation of the immune system by enhancing IL-2 and TNF-a production	2.5, 5, and 7.5 mg/kg/day for 15 days through tail intravenous injection	EGFR-mediated PI3K/AKT and Ras/Raf/MEK/ERK pathway, mitochondria-dependent pathway	Human esophageal cancer cell lines (KYSE-150 and EC9706) and KYSE-150 xenograft mice model	[143]
Se@MIL-101-(P + V) siRNA	MIL-101(Fe), cysteine, Na_2_SeO_3_, siRNA	Spherical, particle size 160 nm, pore diameter 2.19 nm	Enhanced protection of siRNAs against nuclease degradation. Increased siRNA cellular uptake and promoted siRNA escape from endosomes/lysosome to silence MDR genes in MCF-7/T (Taxol-resistance) cells. Enhanced cancer therapeutic efficacy and decreased systemic toxicity in vivo	10 mg/kg by intravenous injection for 15 d (12 μg of siRNA per mouse)	p53, MAPK, and PI3K/Akt	MCF-7/T cells, paclitaxel resistance MCF-7/T cells and nude mice	[144]

BSA: bovine serum albumin; CPT: camptothecin; CTS: chitosan; DOX: doxorubicin; EPI: epirubicin; GSH: glutathione; MDR: multidrug resistance; PPa: pyropheophorbide a; QD: quantum dots; RIS: risedronate sodium; SeNPs: Se-containing nanoparticles.

**Table 3:** Summary of recent work on SeNPs with potential in cancer diagnosis.

SeNP	Material	Shape and Size (nm)	Effects	Dosage	Model	Reference
Anti-HE4 IgG-HE4-anti-HE4^CdSe/ZnS^ immunocomplex	Anti-HE4 IgG antibodies, CdSe/ZnS QD	-	Electrochemical immunosensor for HE4 protein detection using QD as electrochemically active labels of specific antibodies. Contributed significantly to the analytical performance of tumor marker detection and met the exacting requirements for HE4 protein clinical monitoring	-	HE4 in human serum	[145]
Aptamer-modified SeNPs (Apt-SeNPs)	-	Spherical structures, 88 ± 30 nm	Good chemical stability, water solubility, and biocompatibility. Strong green scattering light with a characterized scattering peak at 570 nm. Precisely and specifically target and image nucleolin overexpressed cancer cells after being modified with aptamers	-	Human epidermoid cancer (Hep-2) cells	[146]
CdTe/ZnSe core/shell QDs	CdTe, Na_2_TeO_3_, Zn(CH_3_CO_2_)_2_, Na_2_SeO_3_	QD (10 ± 2 nm), QD+T (13 ± 2 nm), QD+C (18 ± 2 nm), QD+A (71 ± 2 nm), QD+G (95 ± 2 nm)	Able to detect DNAs (directly from cell extracts), damages to the DNA, and mutations	-	Prostate cancer (PC3) and normal human cells (PNT1A)	[147]
IL13 conjugated QD (IL13QD)	CdSe-based QD, 1-ethyl-3-(3-dimethylaminopropyl) carbodiimide (EDC), interleukin-13 (IL13)	Core-shell structure, a size range of 15–20 nm	IL13Rα2 can be detected in cerebrospinal fluid by IL13QD. A higher force of binding interaction between the IL13QD and IL13Rα2 expressing glioma cells and exosomes secreted by glioma stem cells was observed	-	U251 human glioma cells and CD133 positive glioma initiating cells (T3691)	[148]
SeNP loaded imprinted core-shell microcomposites (SIMs)	CTS, zeolite, TiO_2_, Na_2_SeO_4_, Na_2_SeO_3_	Spherical, average size of 80 nm	Could be used for dot-blot immunoassays for rapid serodiagnosis of human lung cancer. The detection time of the colloidal Se dot test for the progastrin-releasing peptide (as a tumor marker for small cell lung cancers) was only 5 min	Linear with the concentration of antigen within the concentration range of 0–105 pg/mL. The lowest concentration to distinguish significant positive results was observed to be 75 pg/mL	Human progastrin releasing-peptide	[149]

CTS: chitosan; HE4: human epididymis protein 4; QD: quantum dots; SeNPs: Se-containing nanoparticles.

**Table 4:** Summary of recent work on multi-functional SeNPs with potential in cancer-related research.

SeNPs	Material	Shape and Size (nm)	Function ^1^	Effects	Dosage	Pathway	Model	Reference
Ag_2_Se-cetuximab nanoprobes	Bis(trimethylsilyl)selenide, silver acetate, cetuximab	Spherical, diameter of 2.8 ± 0.5 nm	C and E	Displayed faster and more enrichment at the site of cancer. Inhibited the tumor growth and improved the survival rate of the cancer-bearing nude mice model. Combined targeted imaging and therapy	-	-	Human tongue squamous cell carcinoma cells (CAL-27) and human immortalized noncancerous keratinocytes cells (HaCaT) and Balb/c mice	[150]
DOX-loaded selenopolymeric nanocarriers (Se@CMHA-DOX NPs)	Na_2_SeO_3_, ascorbic acid, poly (ethylene glycol) (PEG), cetyl-modified hyaluronic acid, DOX	Spherical, 244 ± 6.8 nm	A and C	Inhibited TrxR activity and augmented the anticancer efficacy of DOX. Induced G2/M cell cycle arrest and TP53-mediated caspase-independent apoptosis. Reduced tumor activity in a three-dimensional tumor sphere model	5 μg/mL for 48 h	Apoptotic pathway	MCF7 breast adenocarcinoma cells and MCF7 tumor sphere model	[151]
HSAMSe@DOX	Na_2_SeO_3_, L-ascorbic acid, Human serum albumin, DOX	Homogeneous spherical, ∼80 nm	A and C	Synergistically enhanced the antitumor activity of DOX and decreased the side effects associated with DOX. Increased tumor-targeting effects and enhanced cellular uptake through nanoparticle interact with SPARC protein	10 mg/mL, 100 μL into the veins of the tails	-	MCF-7, MCF-10A, MDA-MB-231, SKBR3 and female BALB/C nude mice	[152]
MoSe_2_(Gd^3+^)-PEG nanosheets	NaMoO_4_·2H_2_O, Se, NaBH_4_, gadolinium(III) chloride hexahydrate	Lamellar, 100–150 nm	B, F and G	Able to provide a strong contrast for *T*1 weighted magnetic resonance imaging. Could be used as contrast agent for photoacoustic imaging (PAI). Increased the temperature to help kill cancer cells under laser irradiation. Enhanced permeation and retention effect in the tumor using magnetic resonance photoacoustic bimodal imaging in vivo. Suppressed tumors in mice by injection with laser irradiation	-	-	Hep G2 human hepatoma carcinoma cells, BALB/c nude mice	[153]
Paclitaxel-loaded SeNPs	SeO_2_, paclitaxel, ascorbic acid, pluronic F-127	Hydrodynamic diameter, 87 nm, spherical	A and B	Significant antiproliferative activity against cancer cells. G2/M phase arrest in a dose-dependent manner leading to apoptosis. Disruption of mitochondrial membrane potential orchestrated with the induction of reactive oxygen species leading to the activation of caspases	-	MMP, caspases	Lung cells (L-132), cervical cancer cells (HeLa), breast cancer cells, (MCF7), non-small lung carcinoma cells (A549) and colorectal adenocarcinoma cells (HT29)	[154]
PPa@CTX-Se-OA/DSPE-PEG2k	Cabazitaxel, PPa, oleic acid, Se powder, DSPE-PEG2k	Spherical, average diameter of 104.1 ± 3.1 nm	C and D	Light irradiation disassembles the structure of ROS-responsive prodrug nanosystems by cleaving the ROS-responsive linkers to accelerate the release of the parent drug	200 ng/mL for 4 h or 24 h	-	4T1 murine breast cancer cells	[19]
Se/iron oxide nanoparticles (Se:IONP)	Na_2_SeO_3_, acetic acid, CTS, hydrophobic IONP	Spherical in a transmission electron microscope, irregular in a scanning electron microscope, 5–9 nm	A and H	An iron oxide core produced by thermal decomposition, followed by a silane ligand exchange, a CTS coating, and Se decoration. Reduced cancer cell viability	-	-	MB-231 breast cancer cells	[155]
Se-containing hydroxyapatite/alginate (SeHA/ALG) composite granules	(NH_4_)_2_HPO_4_, Na_2_SeO_3_⋅5H_2_O, Ca(NO_3_)_2_⋅4H_2_O, hydroxyapatite, alginate sodium, RIS	Spherical, 1.1–1.5 mm	A and C	Biphasic process of releasing sufficient Se and RIS against osteosarcoma cells	-	-	Human osteoblast-like cell line (Saos-2) and normal human fetal osteoblasts (hFOB 1.19)	[156]
SeNPs-DOX-ICG-RP	L-ascorbic acid, Na_2_SeO_3_, dual-target (RC-12 and PG-6 peptides), loaded with both DOX and ICG	Sphere-like morphology with an average size of 110 nm	B and C	NIR-laser irradiation that raised the temperature of the nanosystem and allowed nanoparticles to decompose and release drugs accurately in the tumor site. Reduced the damage of chemotherapy drugs to normal tissue	-	-	HepG2 and normal L02 cells	[157]
Ultra-small Nano-Se	Na_2_SeO_3_, GSH, BSA	Monodisperse spherical shape with a diameter about 27.5 ± 4.3 nm	A and F	Reinforced the toxic effects of irradiation, leading to a higher mortality rate than either treatment used alone. Induced cell cycle arrest at the G2/M phase and the activation of autophagy. Increased both endogenous and irradiation-induced ROS formation. Improved cancer cell sensitivity to the toxic effects of irradiation	0.15 and 0.3 μg/mL, X-rays (6-MeV, 200 cGy/min)	-	MCF-7 breast carcinoma cells	[158]

^1^ Function, A: chemotherapy; B: photothermal therapy; C: drug delivery; D: photodynamic therapy; E: diagnosis; F: radiosensitizer; G: imaging; H: magnetically-targeted. Abbreviations, BSA: bovine serum albumin; CTS: chitosan; DOX: doxorubicin; GSH: glutathione; ICG: indocyanine green; MMP: mitochondrial membrane potential; PPa: pyropheophorbide a; RIS: risedronate sodium; ROS: reactive oxygen species; SeNPs: Se-containing nanoparticles; TrxR: thioredoxin reductase.

## Abbreviations

D-501036	2,5-bis(5-hydroxymethyl-2-selenienyl)-3-hydroxymethyl-*N*-methylpyrrole
MSA	Methylseleninic acid
MSC	Methylselenocysteine
ncRNA	non-coding RNA
NK	Natural killer
NPC	Nutritional prevention of cancer
PBM	Peripheral blood mononuclear
PTM	Post-translational modification
ROS	Reactive oxygen species
Se	Selenium
SELECT	Selenium and vitamin E cancer prevention trial
SeMet	Selenomethionine
SeNPs	Se-containing nanoparticles
SWOG	Southwest Oncology Group
